# A powerful microbial group association test based on the higher criticism analysis for sparse microbial association signals

**DOI:** 10.1186/s40168-020-00834-9

**Published:** 2020-05-11

**Authors:** Hyunwook Koh, Ni Zhao

**Affiliations:** Department of Biostatistics, Bloomberg School of Public Health, Johns Hopkins University, 615 North Wolfe Street, Office E3622, Baltimore, MD 21205 USA

**Keywords:** Microbiome association studies, Microbial ecology, Adaptive association analysis, Higher criticism, Sparse microbial associations, Phylogenetics

## Abstract

**Background:**

In human microbiome studies, it is crucial to evaluate the association between microbial group (e.g., community or clade) composition and a host phenotype of interest. In response, a number of microbial group association tests have been proposed, which account for the unique features of the microbiome data (e.g., high-dimensionality, compositionality, phylogenetic relationship). These tests generally fall in the class of aggregation tests which amplify the overall group association by combining all the underlying microbial association signals, and, therefore, they are powerful when many microbial species are associated with a given host phenotype (i.e., low sparsity). However, in practice, the microbial association signals can be highly sparse, and this is especially the situation where we have a difficulty to discover the microbial group association.

**Methods:**

Here, we introduce a powerful microbial group association test for sparse microbial association signals, namely, microbiome higher criticism analysis (MiHC). MiHC is a data-driven omnibus test taken in a search space spanned by tailoring the higher criticism test to incorporate phylogenetic information and/or modulate sparsity levels and including the Simes test for excessively high sparsity levels. Therefore, MiHC robustly adapts to diverse phylogenetic relevance and sparsity levels.

**Results:**

Our simulations show that MiHC maintains a high power at different phylogenetic relevance and sparsity levels with correct type I error controls. We also apply MiHC to four real microbiome datasets to test the association between respiratory tract microbiome and smoking status, the association between the infant’s gut microbiome and delivery mode, the association between the gut microbiome and type 1 diabetes status, and the association between the gut microbiome and human immunodeficiency virus status.

**Conclusions:**

In practice, the true underlying association pattern on the extent of phylogenetic relevance and sparsity is usually unknown. Therefore, MiHC can be a useful analytic tool because of its high adaptivity to diverse phylogenetic relevance and sparsity levels. MiHC can be implemented in the R computing environment using our software package freely available at https://github.com/hk1785/MiHC.

## Background

The recent advent of next-generation sequencing has enabled unbiased microbiome profiling for all microbes inhabiting in different organs of the human body. The two major sequencing platforms for microbiome profiling are the targeted polymerase chain reaction amplicons for the 16S ribosomal RNA (rRNA) gene [1, 2] and the shotgun metagenomics for the whole microbial genome [3]. These sequencing platforms produce various types of metagenomic information, such as microbial abundance, gene content, and metabolic capacity [4]. Among those, here we focus on a common type of the microbiome data for the microbial composition with relative abundances and phylogenetic relationships. We also consider the operational taxonomic unit (OTU) as a surrogate of microbial species and the smallest unit of the microbial biomarkers nested in different microbial assemblages (e.g., communities (bacteria, fungi, viruses), upper- or lower-level taxa (phyla, classes, orders, families, genera)). The roles of the microbiome on human health or disease have been intensely studied throughout all different microbial assemblages. For example, the community of bacteria has been primarily studied on the disparity in microbial diversity among different populations (e.g., diseased vs. non-diseased, treatment vs. placebo) [5–8]. While the communities of fungi or viruses have been less studied, they are gaining more and more attention [9, 10]. Moreover, investigators have intensely studied the disparity in microbial taxon composition throughout a breadth of hierarchical taxonomic classifications (e.g., phyla to genera) [11, 12].

Here, we refer, in general, the study on the association between any microbial group (e.g., community or clade) composition and a host phenotype (or any other health/disease-related factor) as a microbial group association study. In response to the popularity of such studies, researchers have proposed a number of microbial group association tests while incorporating the unique features of the microbiome data (e.g., high-dimensionality, compositionality, phylogenetic relationship) into their proposed tests. The most popular approaches are the association tests using *α*- or *β*-diversity indices [5, 6]. *α*-diversity measures within-sample diversity, by which the high-dimensional microbiome information can be projected into a single diversity variable. We can then easily test the association between an *α*-diversity index and a host phenotype using a traditional statistical method (e.g., generalized linear models), or we can jointly consider multiple α-diversity indices and conduct an omnibus microbial diversity association analysis using the adaptive microbiome *α*-diversity-based association test (aMiAD) [13]. On the other hand, *β*-diversity measures between-sample diversity (i.e., dissimilarity or distance), by which the high-dimensional microbiome information can be projected into a full rank similarity matrix via a kernel machine framework [14]. We can then test the association using the ANOVA-type association test known as the permutational multivariate analysis of variance (PERMANOVA) [15–17] or the regression-type association test known as the regression-microbiome regression-based kernel association test (MiRKAT) [14], while they result in a similar performance [14]. Researchers have also proposed diverse microbial group association tests to amplify the overall group association by combining all the underlying microbial association signals (e.g., the microbiome sum of powered score tests (MiSPU) [18]).

All the above tests generally fall in the class of aggregation tests as all the underlying microbial association signals are aggregated into the *α*- or *β*-diversity or the overall group association statistic [19]. Therefore, they are powerful when a large number of OTUs are associated with a host phenotype (i.e., low sparsity) [19]. However, in practice, it is possible that only few OTUs are associated with a host phenotype (i.e., high sparsity), and, as an extreme case, even a single OTU can cause human disease (e.g., a small influx of *Escherichia coli* O157:H7 can cause food poisoning [20]). However, it is questionable if the current methods can powerfully discover the microbial group association for the high sparsity situation. For example, it is so obvious that there is a huge disparity in a variety of host phenotypes between the normal and germ-free mice because of the huge disparity in their microbiomes (i.e., presence vs. absence of microbiome) [21], and, in this low sparsity situation, any of the current methods can powerfully discover the microbial group association with no need for any additional method development. Thus, here we instead move our focus onto the high sparsity situation, in which only a small portion of the OTUs are associated with a host phenotype and the pressing issue of powerfully discovering the disparity in a host phenotype driven by the sparse association signals.

We notice that the group association test, known as higher criticism (HC) test, is powerful at high sparsity levels because its test statistic reflects only the single largest association signal among underlying individual association signals [22]. While the use of the higher criticism test has been extended to genome-wide association studies [23, 24], it has not been well-appreciated for the microbiome group association analysis. This might be because of the unique features of the microbiome data and the resulting need for more sophisticated analysis procedures. Thus, here we further tailor the higher criticism test for microbial group association analysis by incorporating phylogenetic information and modulating sparsity levels, as follows. First, we notice that phylogenetically relevant species share similar genetic components and evolutionary histories and, as a result, they are likely to have similar functional effects on a host phenotype [25]. Thus, to improve power when the OTUs associated with a host phenotype are phylogenetically relevant, we introduce a weighted higher criticism test which gives a higher weight to the OTUs whose phylogenetically relevant OTUs have larger association signals. Second, the original higher criticism test is powerful at a high sparsity level but rapidly loses power as the sparsity level decreases. Thus, to improve power for lower sparsity levels, we introduce a modulated higher criticism test which flexibly reflects the single or multiple largest association signal(s) among underlying individual association signals. In addition, we notice that the Simes test [26] is also powerful at high sparsity levels because it requires only a single strong association signal among underlying individual association signals which is significant even after the multiple testing correction. We heuristically, but not theoretically, found that the Simes test is more powerful at excessively high sparsity levels than the higher criticism test while the Simes test more rapidly loses power as the sparsity level decreases than the higher criticism test (see the “Simulation results” section).

Here, the dilemma in reality is that the OTUs associated with a host phenotype can be phylogenetically relevant or not, and they can be highly sparse or less sparse. Yet, unfortunately, we cannot presume which specific association pattern underlies our study in advance because of the lack of prior knowledge. Thus, here we introduce a data-driven omnibus test, namely, microbiome higher criticism analysis (MiHC), which robustly adapts to diverse association patterns. To achieve the robust adaptivity, we first construct multiple candidate tests by combining the principles of the original, weighted and modulated higher criticism tests, and the Simes test, in which each of the individual candidate tests suits some specific association pattern. Then, we use the minimum *p* value among those candidate tests as the test statistic of MiHC with the aim of closely reaching the highest power among those candidate tests. Finally, we use a residual-based permutation approach based on the minimum *p* value statistic to calculate the *p* value for MiHC. Here, the residual-based permutation approach enables to preserve OTU-by-OTU correlations [27], which are inherent in the microbiome data because of the compositional constraint (also known as unit sum constraint), phylogenetic relevance, and other potential sources.

Our extensive simulations show that MiHC robustly maintains a high power at different phylogenetic relevance and sparsity levels with correct type I error controls at the significance level of 5%. We also apply MiHC to four real microbiome datasets to test the association between respiratory tract microbiome and smoking status [28], the association between the infant’s gut microbiome and delivery mode [29], the association between the gut microbiome and type 1 diabetes status [30], and the association between the gut microbiome and human immunodeficiency virus (HIV) status [31].

## Methods and materials

This section is devoted to describe methodological details (i.e., models, notations, test statistics, and computational procedures) for our proposed methods. Since we use many notations, we organize them in a summary table for easy follow-up (Additional file 1: Table S1).

### Generalized linear models and marginal score statistics

We suppose that the data include *n* samples, *m* OTUs in a microbial group of interest (e.g., community or clade) and *l* covariates (e.g., age, gender). Let *y*_*i*_ denote a host phenotype (or any other health/disease-related factor) of interest, *o*_*ij*_ denote an OTU in relative abundance (i.e., proportion), and *x*_*ik*_ denote a covariate for *i* = 1,…, *n*, *j* = 1,…, *m* and *k* = 1,…, *l*. To test the association between OTUs and a host phenotype adjusting for covariates, we consider a generalized linear model [32] (Eq. 1),

1 $$ g\left({\mu}_i\right)={x}_i^T\alpha +{o}_i^T\beta, $$

where *g*(·) is a canonical link function, *μ*_*i*_ = *E*(*y*_*i*_ ∣ *x*_*i*_, *o*_*i*_), and *α* = (*α*_0_, …, *α*_*l*_)^*T*^ and *β* = (*β*_1_, …, *β*_*m*_)^*T*^ are the regression coefficients for the covariates, *x*_*i*_ = (1, *x*_*i*1_, …, *x*_*il*_)^*T*^, and the OTUs, *o*_*i*_ = (*o*_*i*1_, …, *o*_*im*_)^*T*^, respectively. Here, *y*_*i*_ conditional on *x*_*i*_ and *o*_*i*_ is assumed to follow a distribution in the exponential dispersion family with the probability density/mass function (Eq. 2).

2 $$ f\left({y}_i|\ {\theta}_i,\varphi \right)=\exp \left\{\frac{y_i{\theta}_i-b\left({\theta}_i\right)}{a_i\left(\varphi \right)} + c\left({y}_i,{\theta}_i\right)\right\}, $$

where *θ*_*i*_ is the natural parameter, *φ* is the dispersion parameter, and *a*(·), *b*(·), and *c*(·) are the known functions [32]. Let *b*^′^(*θ*_*i*_) and *b*^′′^(*θ*_*i*_) denote the first two derivatives of *b*(*θ*_*i*_) evaluated at *θ*_*i*_; as such, *E*(*y*_*i*_ ∣ *x*_*i*_, *o*_*i*_) = *b*^′^(*θ*_*i*_) and Var(*y*_*i*_ ∣ *x*_*i*_, *o*_*i*_) = *a*_*i*_(*φ*)*b*^′′^(*θ*_*i*_). Here, we are interested in testing the global null hypothesis of no association between OTUs and a host phenotype adjusting for covariates (Eq. 3).

3 $$ {\displaystyle \begin{array}{c}{H}_0:{\beta}_j=0\mathrm{forall}{j}^{\prime}\sin \left\{1,\dots, m\right\}\mathrm{vs}.\kern0.5em \\ {}\kern0ex {H}_1:{\beta}_j\ne 0\mathrm{forsome}{j}^{\prime}\sin \left\{1,\dots, m\right\}\end{array}} $$

While we will soon address the above global hypothesis testing in the following sections, here we first delineate the marginal standardized score statistic for each OTU (Eq. 4) as it is the key component of the higher criticism test [24].

4 $$ {Z}_j=\frac{o_j^T\left(y-{\hat{\mu}}_0\right)}{\sqrt{o_j^TP{o}_j}}, $$

where *o*_*j*_ = (*o*_1*j*_, …, *o*_*nj*_)^*T*^, *y* = (*y*_1_, …, *y*_*n*_)^*T*^, ${\hat{\mu}}_0$ is the vector of the expected values of *y*_*i*_’s estimated under the null model of *g*(*μ*_*i*_) = ${x}_i^T\alpha$; ${\hat{\mu}}_0$ = ${\left({\hat{\mu}}_{1,0},\dots, {\hat{\mu}}_{n,0}\right)}^T$ = ${\left({g}^{-1}\left({x}_1^T{\hat{\alpha}}_0\right),\dots, {g}^{-1}\left({x}_n^T{\hat{\alpha}}_0\right)\right)}^T$ = ${\left({b}^{\prime}\left({\hat{\theta}}_{1,\kern0.5em 0}\right),\dots, {b}^{\prime}\left({\hat{\theta}}_{n,\kern0.5em 0}\right)\right)}^T$, and *P* = *W* − *WX*(*X*^*T*^*WX*)^−1^*X*^*T*^*W*, where *W* is the diagonal matrix of the marginal variances of *y*_*i*_’s estimated under the null model of *g*(*μ*_*i*_) = ${x}_i^T\alpha$; *W =* diag(*a*_1_(${\hat{\varphi}}_0\Big){b}^{\prime \prime}\left({\hat{\theta}}_{1,\kern0.5em 0}\right)$, …, *a*_*n*_(${\hat{\varphi}}_0\Big){b}^{\prime \prime}\left({\hat{\theta}}_{n,\kern0.5em 0}\right)$), and *X* = (*x*_1_, …, *x*_*n*_)^*T*^, for *j* = 1,…, *m*. Here, the statistic *Z*_*j*_ tells the effect direction and size for the *j*th OTU, and we assume that *Z*_*j*_ follows the standard normal distribution *N*(0,1) under the marginal null hypothesis of *β*_*j*_ = 0. Then, we can calculate the marginal *p* value for the *j*th OTU as *P*(|*Z*_*j*_| > *N*(0,1)).

### Unweighted and weighted higher criticism analyses

Donoho and Jin first derived the higher criticism test, motivated by an idea of the great statistician, John Wilder Tukey [22]. Then, the higher criticism test has been further developed by a few follow-up studies [23, 24, 33, 34]. While there are different forms of the test statistic, we use the simplest form of (Eq.5) based on [23].

5 $$ \mathrm{uHC}={\max}_{j\in \left\{1,\dots, m\right\}}\left\{\frac{r_j/m-{p}_j}{\sqrt{p_j\left(1-{p}_j\right)/m}}\right\}, $$

where uHC is the test statistic for the higher criticism test [23], *p*_*j*_ is the *p* value for the *j*th OTU, and *r*_*j*_ is the rank of *p*_*j*_ in the ascending order of *p*_*j*_’s for *j* = 1,…, *m*. We denote this higher criticism test as the unweighted higher criticism (uHC) test in order to distinguish it from the forthcoming weighted higher criticism test. Here, a relatively large observed statistic value compared with null statistic values indicates a higher chance to discover the group association. Prior studies have found that this higher criticism test sensitively detects highly sparse association signals [22–24, 33]. The major rationale behind is that the test statistic (Eq. 5) focuses on the single largest deviation between the expected ($\frac{r_j/m}{\sqrt{p_j\left(1-{p}_j\right)/m}}$) and observed ($\frac{p_j}{\sqrt{p_j\left(1-{p}_j\right)/m}}$) quantiles of significance among all the *m* tests; as such, only a small number of association signals are sufficient to get a large statistic value [22–24].

In microbiome association studies, phylogenetically relevant species tend to have similar effects on a host phenotype because of their similarities in genetic components and evolutionary histories [25]. Thus, to improve power when the OTUs associated with a host phenotype are phylogenetically relevant, we introduce the weighted higher criticism (wHC) test (Eq. 6).

6 $$ \mathrm{wHC}={\max}_{j\in \left\{1,\dots, m\right\}}\left\{\frac{w_j\left({r}_j/m-{p}_j\right)}{\sqrt{p_j\left(1-{p}_j\right)/m}}\right\}, $$

where wHC is the test statistic for the weighted higher criticism test and *w*_*j*_ is the weight for the *j*th OTU. To assign the weight to each OTU (i.e., *w*_*j*_ for *j* = 1,…, *m*), we first partition the *m* OTUs into *C* phylogenetically close clusters based on OTUs’ pairwise cophenetic distances [35], where the cophenetic distance of any two OTUs refers to the total length of the branches to their most common ancestor (i.e., the closest intersection) in the phylogenetic tree and we calculate it using the function, *cophenetic*, in the R package, *stats*. For this, we use the partitioning-around-medoids algorithm [36] based on the optimal number of clusters (*C*) which maximizes the average silhouette width searching up to 30 clusters [36]. Let ζ(*j*) denote a cluster anchored at the *j*th OTU among the *C* clusters. Then, we define *w*_*j*_ as (Eq. 7),

7 $$ {w}_j=\frac{\sum \limits_{j^{\prime}\in \upzeta (j)\backslash \left\{j\right\}}\ \frac{1}{D_{j,{j}^{\prime }}}\mid {Z}_{j^{\prime }}\mid }{\sum \limits_{j^{\prime}\in \upzeta (j)\backslash \left\{j\right\}}\ \frac{1}{D_{j,{j}^{\prime }}}}+1, $$

where ${D}_{j,{j}^{\prime }}$ is the cophenetic distance between *j*th and *j*^′^th OTUs, *j* ∈ ζ(*j*) and *j*^′^ ∈ ζ(*j*)\{*j*}. *w*_*j*_ is designed to give a higher weight to the OTUs whose neighboring OTUs, with respect to closer phylogeny (see $\frac{1}{D_{j,{j}^{\prime }}}$), have larger association signals (see $\mid {Z}_{j^{\prime }}\mid$). Therefore, *w*_*j*_ amplifies the association signals from close phylogeny and hence can suit when the OTUs associated with a host phenotype are phylogenetically relevant.

### Modulated higher criticism analyses for lower sparsity levels

Again, the higher criticism test is powerful for high sparsity levels, but it is underpowered for low sparsity levels [24]. In practice, the true associations are not always so highly sparse that the higher criticism can be underpowered. Thus, to improve power for lower sparsity levels, we make some modulations to the original test statistic as (Eq. 8),

8 $$ {\mathrm{uHC}}_{(h)}=\frac{1}{h}\sum \limits_{j^{\prime }=1}^h\frac{r_{j^{\prime }}/m-{p}_{j^{\prime }}}{\sqrt{p_{j^{\prime }}\left(1-{p}_{j^{\prime }}\right)/m}}, $$

where uHC_(*h*)_ is the test statistic for the unweighted higher criticism test for a given *h* value, $\frac{r_{j^{\prime }}/m-{p}_{j^{\prime }}}{\sqrt{p_{j^{\prime }}\left(1-{p}_{j^{\prime }}\right)/m}}$ is the *j*^′^th order statistics of $\frac{r_j/m-{p}_j}{\sqrt{p_j\left(1-{p}_j\right)/m}}$’s in the descending order for *j* = 1,…, *m*, and *h* needs to be pre-specified, *h* ∈ {1, 2, …, *m*-1, *m*}. uHC_(*h*)_ is the average of the first *h* largest deviations between the expected ($\frac{r_j/m}{\sqrt{p_j\left(1-{p}_j\right)/m}}$) and observed ($\frac{p_j}{\sqrt{p_j\left(1-{p}_j\right)/m}}$) quantiles of significance among all the *m* tests (Eq. 8). uHC_(*h*)_ is also a generalization of the original higher criticism test (Eq. 5) because when *h* = 1, uHC_(*h*)_ becomes the original higher criticism test (i.e., uHC_(1)_). uHC_(1)_ relies on the single largest deviation and hence can suit high sparsity levels. As *h* increases, uHC_(*h*)_ considers more deviations to the next level association signals and hence can suit lower sparsity levels. When *h*=*m*, uHC_(*h*)_ becomes uHC_(*m*)_. uHC_(*m*)_ considers all the *m* deviations and hence can suit the least sparsity level. Without loss of generality, we can apply the same modulations to the weighted higher criticism (wHC) test (Eq. 9).

9 $$ {\mathrm{wHC}}_{(h)}=\frac{1}{h}\sum \limits_{j^{\prime }=1}^h\frac{w_{j^{\prime }}\left({r}_{j^{\prime }}/m-{p}_{j^{\prime }}\right)}{\sqrt{p_{j^{\prime }}\left(1-{p}_{j^{\prime }}\right)/m}}, $$

where wHC_(*h*)_ is the test statistic for the weighted higher criticism test for a given *h* value, and $\frac{w_{j^{\prime }}\left({r}_{j^{\prime }}/m-{p}_{j^{\prime }}\right)}{\sqrt{p_{j^{\prime }}\left(1-{p}_{j^{\prime }}\right)/m}}$ is the *j*^′^th order statistics of $\frac{w_j\left({r}_j/m-{p}_j\right)}{\sqrt{p_j\left(1-{p}_j\right)/m}}$’s in the descending order for *j* = 1,…, *m*. We calculate the *p* values for the individual unweighted (uHC_(*h*)_’s) and weighted (wHC_(*h*)_’s) tests based on a permutation method (see the “*p* value calculation” section).

### Simes test

Simes (1986) introduced a simple modification of the Bonferroni procedure for multiple hypothesis testing and a group association test, known as the Simes test, that calculates the *p* value as the minimum *p* value among the marginal *p* values that are corrected by the Bonferroni procedure (i.e., multiplied by the number of tests) and weighted by the inverse of their ranks (i.e., multiplied by the inverse of their ranks) [26] (Eq. 10).

10 $$ {P}_{\mathrm{Simes}}={T}_{\mathrm{Simes}}={\min}_{j\in \left\{1,\dots, m\right\}}\left\{\frac{m{p}_j}{r_j}\right\}, $$

where *P*_Simes_ and *T*_Simes_ are the *p* value and the test statistic for the Simes test, *p*_*j*_ is the *p* value for the *j*th OTU, and *r*_*j*_ is the rank of *p*_*j*_ in the ascending order of *p*_*j*_’s for *j* = 1,…, *m*. To discover the group association, the Simes test requires only a single strong association signal which can produce a significant *p* value even after adjusting for multiple hypothesis testing. Thus, the Simes test is also powerful at highly sparsity levels. Our simulations demonstrate that the Simes test is more powerful at excessively high sparsity levels than the higher criticism test while the Simes test more rapidly loses power as the sparsity level decreases (see the “Simulation results” section).

### Microbiome higher criticism analysis

In reality, the true microbial associations can be phylogenetically relevant or not, and they can be highly sparse or less sparse, yet we do not know the true underlying association pattern in advance. Thus, to robustly adapt to the unknown phylogenetic relevance and sparsity levels, we propose a data-driven omnibus test, namely, microbiome higher criticism (MiHC) analysis (Eq. 11).

11 $$ {T}_{\mathrm{MiHC}}=\min \left(\underset{h\upepsilon \Gamma}{\min}\left({P}_{\mathrm{uHC}(h)},{P}_{\mathrm{wHC}(h)}\right),{P}_{\mathrm{Simes}}\right), $$

where *P*_uHC(*h*)_’s are the *p* values based on the uHC_(*h*)_ tests, *P*_wHC(*h*)_’s are the *p* values based on the wHC_(*h*)_ tests for *h*’s in a set Г (e.g., Г = {1, 3, 5, 7, 9}), and *P*_Simes_ is the *p* value based on the Simes test. *T*_MiHC_ is the minimum *p* value among all the uHC_(*h*)_ (Eq. 8) and wHC_(*h*)_ (Eq. 9) tests for *h*’s in Г and the Simes test (Eq. 10). Of course, we do not use this minimum *p* value as the final *p* value for MiHC, but we instead use it as the test statistic of MiHC. We calculate the *p* value for MiHC based on a permutation method (see the “*p* value calculation” section). This kind of the minimum *p* value statistic approach has also been widely used in many prior association tests [13, 14, 18, 37–39]. The set (Г) can be spanned up to the union set of {1, 2,…, *m*-1, *m*}. However, it is a huge computational burden to survey all the *h* values in the union set because of the high-dimensionality of the microbiome data. Thus, we use a candidate set of Г = {1, 3, 5, 7, 9} and it was sufficient in our simulations and real data applications. The use of the minimum *p* value statistic allows MiHC to closely approach the most powerful test among all the candidate tests in Г and the Simes test. Therefore, compared with the original higher criticism test (which is only for *h* = 1) or the Simes test, our candidate set always gives a similar or higher power. Our extensive simulation experiments demonstrate the high adaptivity of MiHC to various phylogenetic relevance and sparsity levels while robustly maintaining a high power with well-controlled type I error rates (see the “Simulation results” section).

By the same logic, we can also consider two local omnibus tests, namely, uHC_*A*_ (Eq. 12) and wHC_*A*_ (Eq. 13), that are taken within each of the two sub-domains: (1) the unweighted higher criticism tests (i.e., uHC_(*h*)_tests for *h*’s in Г) and the Simes test and (2) the weighted higher criticism tests (i.e., wHC_(*h*)_ tests for *h*’s in Г) and the Simes test.

12 $$ {T}_{{\mathrm{uHC}}_A}=\min \left(\underset{h\upepsilon \Gamma}{\min}\left({P}_{\mathrm{uHC}(h)}\right),{P}_{\mathrm{Simes}}\right), $$

13 $$ {T}_{{\mathrm{wHC}}_A}=\min \left(\underset{h\upepsilon \Gamma}{\min}\left(\ {P}_{\mathrm{wHC}(h)}\right),{P}_{\mathrm{Simes}}\right), $$

${T}_{{\mathrm{uHC}}_A}$ is the minimum *p* value among uHC_(*h*)_ (Eq. 8) tests and the Simes test (Eq. 10) while ${T}_{{\mathrm{wHC}}_A}$ is the minimum *p* value among wHC_(*h*)_ (Eq. 9) tests and the Simes test (Eq. 10). These two local omnibus tests are distinguished from the global omnibus test, MiHC, that is taken within the global domain of all the unweighted and weighted higher criticism tests (i.e., all the uHC_(*h*)_ and wHC_(*h*)_ tests for *h*’s in Г) and the Simes test. ${T}_{{\mathrm{uHC}}_A}$ and ${T}_{{\mathrm{wHC}}_A}$ are the test statistics of uHC_*A*_ and wHC_*A*_, respectively, and we calculate the *p* value based on a permutation method (see the “*p* value calculation” section). By the formula, we can infer that uHC_*A*_ and wHC_*A*_ can modulate sparsity levels through *h*’s in Г and the Simes test for excessively high sparsity levels, while uHC_*A*_ suits the low phylogenetic relevance, but wHC_*A*_ suits the high phylogenetic relevance. Although the global omnibus test (i.e., MiHC) (Eq. 11) is our major proposal for microbial group association analysis, we introduce these two local omnibus tests (i.e., uHC_*A*_ and wHC_*A*_) especially because uHC_*A*_ is useful to modulate sparsity levels when the phylogenetic information is not available (e.g., microbial functional studies for genetic/metabolic content).

### *p* value calculation

There have been different approaches to calculate the *p* value for the higher criticism test [22–24, 33, 34]. The analytical approaches based on an asymptotic distribution proposed in [22, 33, 34] have the advantage of producing a closed-form *p* value in a computationally efficient manner. However, the analytical approaches assume independent tests and/or rely on asymptotics in *m* which requires *m* as large as a million for valid statistical inferences [23]. In microbiome association studies, the independence assumption can be easily violated because of the inherent compositional constraint and phylogenetic relevance. Furthermore, the microbiome data do not usually include a million OTUs so that the slow convergence rate to asymptotics in *m* can lead to invalid statistical inferences [23]. Thereafter, Barnett et al. proposed an exact *p* value calculation which releases the independence assumption and the huge *m* requirement. However, its computational burden increases exponentially as *m* increases; hence, it can handle only a small number of OTUs. Therefore, instead of using the asymptotic or the exact method, we use a permutation method to calculate the *p* value for our proposed method. In particular, we use the following procedures.

1. Fit the null generalized linear model and estimate the residuals as ${\hat{e}}_0$ = ${\left({y}_1-{g}^{-1}\left({x}_1^T{\hat{\alpha}}_0\right),\dots, {y}_n-{g}^{-1}\left({x}_n^T{\hat{\alpha}}_0\right)\right)}^T$.
2. Calculate the marginal score statistics as *Z*_*j*_ = ${o}_j^T{\hat{e}}_0/\sqrt{o_j^TP{o}_j}$ (Eq. 4) and the marginal *p* values as *p*_*j*_ = *P*(|*Z*_*j*_| > *N*(0,1)) for *j* = 1,…, *m*. Calculate the observed statistics, uHC_(*h*)_ (Eq. 8) and wHC_(*h*)_ (Eq. 9), for each *h* ∈ Г, and the *p* value for the Simes test, *P*_Simes_ (Eq. 10).
3. Permute the estimated residuals ${\hat{e}}_0$ multiple times (say, *B* times) and denote each permuted residual vector as ${e}_b^{\prime }$ for *b* = 1,…, *B*. Repeat step 2 *B* times, replacing ${\hat{e}}_0$ with each ${e}_b^{\prime }$, and calculate the null statistics, uHC_(*h*)(*b*)_ (Eq. 8) and wHC_(*h*)(*b*)_ (Eq. 9), for each *h* ∈ Г and for each *b* ∈ {1,…, *B*} and the null statistics for the Simes test, *T*_Simes(*b*)_ = ${\min}_{j\in \left\{1,\dots, m\right\}}\left\{\frac{m{p}_{j(b)}}{r_{j(b)}}\right\}$ for each *b* ∈ {1,…, *B*}.
4. Calculate *P*_uHC(*h*)_ = $\sum \limits_{b=1}^B\left[I\right({\mathrm{uHC}}_{(h)(b)}$ > uHC_(*h*)_)+1]/(*B*+1) and *P*_wHC(*h*)_ = $\sum \limits_{b=1}^B\left[I\right({\mathrm{wHC}}_{(h)(b)}$ > wHC_(*h*)_)+1]/(*B*+1) for each *h* ∈ Г. Calculate the observed statistics, ${T}_{{\mathrm{uHC}}_A}$ = $\min \left(\underset{h\upepsilon \Gamma}{\min}\left({P}_{\mathrm{uHC}(h)}\right),{P}_{\mathrm{Simes}}\right)$ (Eq. 12), ${T}_{{\mathrm{wHC}}_A}$ = $\min \left(\underset{h\upepsilon \Gamma}{\min}\left({P}_{\mathrm{wHC}(h)}\right),{P}_{\mathrm{Simes}}\right)$ (Eq. 13) and *T*_MiHC_ = ${T}_{{\mathrm{MiHC}}} = \min \left(\underset{h\epsilon}{\min}\Gamma\,(P_{\text{uHC}(h)}),(P_{\text{wHC}(h)}), P_{\text{Simes}}\right)$ (Eq. 11).
5. Calculate *P*_uHC(*h*)(*b*)_ =${\sum}_{b^{\prime}\ne b}\left[I\left({\mathrm{uHC}}_{(h)\left({b}^{\prime}\right)}>{\mathrm{uHC}}_{(h)(b)}\right)+1\right]$/(*B*+1) and *P*_wHC(*h*)(*b*)_ = ${\sum}_{b^{\prime}\ne b}\left[I\left({\mathrm{wHC}}_{(h)\left({b}^{\prime}\right)}>{\mathrm{wHC}}_{(h)(b)}\right)+1\right]$/(*B*+1) for each *h* ∈ Г, and *P*_Simes(*b*)_ =${\sum}_{b^{\prime}\ne b}\left[I\left({T}_{\mathrm{Simes}\left({b}^{\prime}\right)}<{T}_{\mathrm{Simes}(b)}\right)+1\right]$/(*B*+1), where *b* ∈ {1,…, *B*} and *b*^′^ ∈ {1,…, *B*}. Calculate the null statistics, ${T}_{{\mathrm{uHC}}_{A(b)}}$ = $\min \left(\underset{h\upepsilon \Gamma}{\min}\left({P}_{\mathrm{uHC}(h)(b)}\right),{P}_{\mathrm{Simes}(b)}\right)$ (Eq. 12), ${T}_{{\mathrm{wHC}}_{A(b)}}$ = $\min \left(\underset{h\upepsilon \Gamma}{\min}\left({P}_{\mathrm{wHC}(h)(b)}\right),{P}_{\mathrm{Simes}(b)}\right)$ (Eq. 13) and *T*_MiHC(*b*)_ = $\min \left(\underset{h\upepsilon \Gamma}{\min}\left({P}_{\mathrm{uHC}(h)(b)},{P}_{\mathrm{wHC}(h)(b)}\right),{P}_{\mathrm{Simes}(b)}\right)$ (Eq. 11), for *b* = 1,…, *B*.
6. Calculate the *p* values for uHC_*A*_ as ${P}_{{\mathrm{uHC}}_A}=\sum \limits_{b=1}^B\left[I\right({T}_{{\mathrm{uHC}}_A(b)}$ < ${T}_{{\mathrm{uHC}}_A}$) + 1]/(*B*+1), wHC_*A*_ as ${P}_{{\mathrm{wHC}}_A}=\sum \limits_{b=1}^B\left[I\right({T}_{{\mathrm{wHC}}_A(b)}$ < ${T}_{{\mathrm{wHC}}_A}$) + 1]/(*B*+1) and MiHC as *P*_MiHC_ = $\sum \limits_{b=1}^B\left[I\right({T}_{\mathrm{MiHC}(b)}$ < *T*_MiHC_) + 1]/(*B*+1).

Importantly, our permutation method can robustly account for any correlation structure among the *m* tests using the same permuted residual vectors repeatedly for each test (i.e., residual-based permutation) [27]. Moreover, since MiHC is based on the score test (Eq. 4) which is computationally efficient and the null model needs to be fitted only once, our method is computationally manageable.

### Visualization

Here, we introduce simple Q-Q plots to demonstrate influential OTUs in each of the two sub-domains (i.e., uHC and wHC) for MiHC. First, we draw Q-Q plots between the expected ($\frac{r_j/m}{\sqrt{p_j\left(1-{p}_j\right)/m}}$) and observed ($\frac{p_j}{\sqrt{p_j\left(1-{p}_j\right)/m}}$) quantiles for uHC (Eq. 5) and between the expected ($\frac{w_j\left({r}_j/m\right)}{\sqrt{p_j\left(1-{p}_j\right)/m}}$) and observed ($\frac{w_j{p}_j}{\sqrt{p_j\left(1-{p}_j\right)/m}}$) quantiles for wHC (Eq. 6), respectively, for *j* = 1,…, *m*. Here, we use (blue) dots to represent individual OTUs and a (red) diagonal line with intercept 0 and slope 1 to represent no influential points; as such, the OTUs that fall along the diagonal line have no influence on the host phenotype while the OTUs that have larger deviations from the diagonal line are more influential on the host phenotype. Then, we report the 10 most influential OTUs corresponding to the 10 largest deviations from the diagonal line with respect to uHC and wHC, respectively. We use darker to lighter vertical lines to represent more to less influential OTUs in rank order among the 10 most influential OTUs. Example visualizations can be found later in the “Real data applications” section.

### Simulation results

We conducted simulation experiments to compared MiHC with the prior tests, Simes test [26], higher criticism (HC) test (i.e., uHC (Eq. 5)) [22], aMiAD [13], adaptive MiSPU (aMiSPU) [18], and Optimal MiRKAT (OMiRKAT) [14]. Our simulation design is based on prior studies [14]. We first estimated the proportions and dispersion for the 100 most abundant OTUs from the real respiratory tract microbiome data [28] based on the Dirichlet-multinomial model [40]. Then, we iteratively generated an OTU count table using the Dirichlet-multinomial model with the estimated proportions and dispersion and a rooted phylogenetic tree with 100 leaves using the function, *rtree*, in the R package, *ape* [41]. Here, we fixed the total reads per sample as 1000 to mimic the compositional constraint and considered two different sample sizes, *n* = 50 and *n* = 100, respectively. To illustrate fits of the simulated data, we generated histograms of the relative abundances for the 100 most abundant OTUs of the real respiratory tract microbiome data and the simulated data based on the Dirichlet-multinomial model (Additional file 2: Figure S1). For the relative abundances of the simulated data, we took averages across 100 simulated data sets that were iteratively generated based on the Dirichlet-multinomial model for *n* = 50 and *n* = 100, respectively. To estimate type I error rates and powers, we generated Gaussian responses based on the linear regression model below.

$$ {y}_i=0.5\times \mathrm{scale}\left({x}_{1i}\right)+0.5\times \mathrm{scale}\left({x}_{2i}\right)+\beta \times \sum \limits_{j\in \Lambda}\mathrm{scale}\left({o}_{ij}\right)+{\varepsilon}_i, $$

where *x*_*i*1_ are *x*_*i*2_ are the covariates generated from the Bernoulli distribution with success probability 0.5 and the standard normal distribution *N*(0, 1), respectively, Λ is a set of OTUs that are associated with the host phenotype *y*_*i*_, *ε*_*i*_ is an error term generated from the standard normal distribution *N*(0, 1), and scale is the standardization function to have mean 0 and standard deviation 1.

To estimate type I error rates, we assigned *β* = 0 to reflect the null hypothesis of no association for all OTUs (Eq. 3). To estimate powers, we assigned *β* = 1 for *n* = 50 and *β* = 0.5 for *n* = 100, while choosing the set of associated OTUs (Λ) based on two different scenarios: (1) we randomly selected 2%, 4%, 6%, 8%, 10%, or 12% of the OTUs to be associated with the host phenotype and (2) we selected 2%, 4%, 6%, 8%, 10%, or 12% of the OTUs which are phylogenetically close to be associated with the host phenotype. We regard the second scenario more realistic because the phylogenetic relevance likely to give shared functional attributes. In particular, for the second scenario, we randomly selected one OTU as a seed OTU and then included 2%, 4%, 6%, 8%, 10%, or 12% of the OTUs that are closest to the seed OTU (including the seed OTU) with respect to cophenetic distance [35]. For both of the scenarios, 2%, 4%, 6%, 8%, 10%, and 12% reflect from high to low sparsity levels.

## Results

### Simulation results

#### Fits of the simulated data

Additional file 2: Figure S1 reports the histograms of the relative abundances for the real respiratory tract microbiome data [28] and the simulated data based on the Dirichlet-multinomial model [40]. We can observe that the simulated data approximate to the real data in shape while including high proportions for rare OTUs (Additional file 2: Figure S1). This indicates that the Dirichlet-multinomial model is useful to simulate microbiome data.

#### Type I error

Table 1 reports empirical type I error rates at the significance level of 5% for all the surveyed methods. We can observe correct type I error controls (i.e., the empirical type I error rates close to the significance level of 5%) for all the individual (i.e., uHC_(*h*)_’s and wHC_(*h*)_’s) and omnibus (i.e., uHC_*A*_, wHC_*A*_, and MiHC) higher criticism tests and the Simes test and also for all the other competing tests (i.e., aMiAD, aMiSPU, and OMiRKAT) (Table 1). Therefore, all the surveyed tests are valid in hypothesis testing.

**Table 1:** Empirical type I error rates at the significance level of 5% for the individual (i.e., uHC_(*h*)_’s and wHC_(*h*)_’s for *h* ∈ {1, 3, 5, 7, 9}) and omnibus (i.e., uHC_*A*_, wHC_*A*_, and MiHC) higher criticism tests, the Simes test, and the other competing tests (i.e., aMiAD, aMiSPU, and OMiRKAT)

Category		Method	*n*=50	*n*=100
Individual HC tests	Unweighted tests	uHC_(1)_	0.051	0.049
		uHC_(3)_	0.050	0.052
		uHC_(5)_	0.049	0.049
		uHC_(7)_	0.050	0.050
		uHC_(9)_	0.050	0.051
	Weighted tests	wHC_(1)_	0.047	0.047
		wHC_(3)_	0.048	0.049
		wHC_(5)_	0.048	0.049
		wHC_(7)_	0.049	0.049
		wHC_(9)_	0.049	0.049
Omnibus HC tests	Local omnibus tests	uHC_*A*_	0.049	0.049
		wHC_*A*_	0.049	0.049
	Global omnibus tests	MiHC	0.050	0.052
Non-HC tests		Simes	0.048	0.049
		aMiAD	0.050	0.051
		aMiSPU	0.051	0.051
		OMiRKAT	0.050	0.050

#### Power

**Fig. 1:**
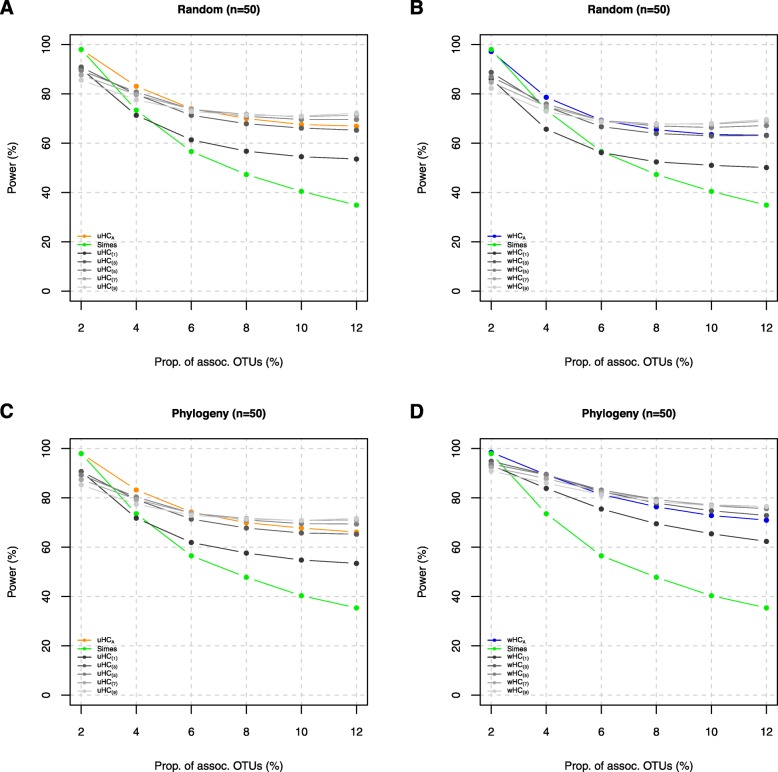
Power estimates for the individual (i.e., uHC_(*h*)_’s and wHC_(*h*)_’s for *h* ∈ {1, 3, 5, 7, 9}) and local omnibus (uHC_*A*_ and wHC_*A*_) higher criticism tests (*n* = 50) (unit, %). **a** uHC_(*h*)_’s and uHC_*A*_ for the randomly selected OTUs (i.e., Λ = {2%, 4%, 6%, 8%, 10% or 12% random OTUs}). **b** wHC_(*h*)_’s and wHC_*A*_ for the randomly selected OTUs (i.e., Λ = {2%, 4%, 6%, 8%, 10% or 12% phylogenetically close OTUs}). **c** uHC_(*h*)_’s and uHC_*A*_ for the phylogenetically relevant OTUs (i.e., Λ = {2%, 4%, 6%, 8%, 10% or 12% random OTUs}). **d** wHC_(*h*)_’s and wHC_*A*_ for the phylogenetically relevant OTUs (i.e., Λ = {2%, 4%, 6%, 8%, 10% 12% phylogenetically close OTUs})

**Fig. 2:**
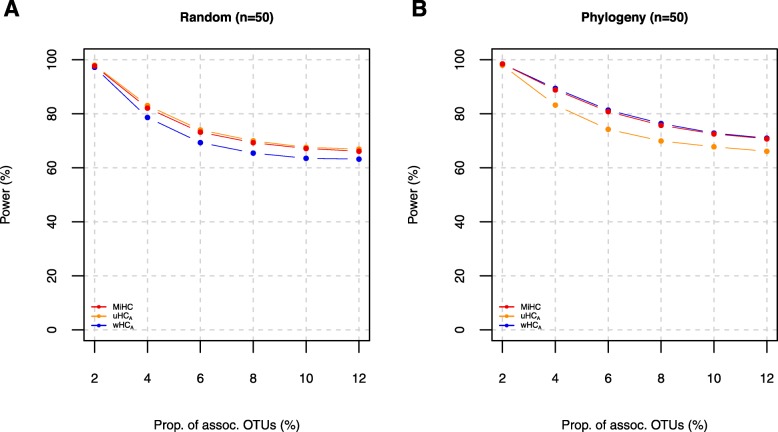
Power estimates for the omnibus (uHC_*A*_, wHC_*A*_, and MiHC) higher criticism tests (*n* = 50) (unit, %). **a** For the randomly selected OTUs (i.e., Λ = {2%, 4%, 6%, 8%, 10% or 12% random OTUs}. **b** For the phylogenetically relevant OTUs (i.e., Λ = {2%, 4%, 6%, 8%, 10% or 12% phylogenetically close OTUs})

**Fig. 3:**
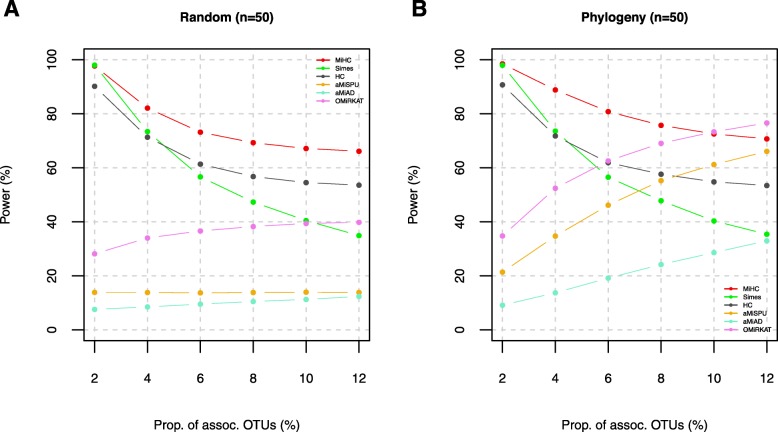
Power estimates for MiHC compared with the prior tests, HC, aMiAD, aMiSPU, and OMiRKAT (*n* = 50) (unit, %). **a** For the randomly selected OTUs (i.e., Λ = {2%, 4%, 6%, 8%, 10% or 12% random OTUs}. **b** For the phylogenetically relevant OTUs (i.e., Λ = {2%, 4%, 6%, 8%, 10% or 12% phylogenetically close OTUs})

Here, we report the power comparisons in the order of (i) the comparison for the individual (i.e., uHC_(*h*)_’s and wHC_(*h*)_’s) and local omnibus (i.e., uHC_*A*_ and wHC_*A*_) higher criticism tests and the Simes test (Fig. 1 (*n* = 50) and Additional file 3: Figure S2 (*n* = 100)); (ii) the comparison for the local omnibus (i.e., uHC_*A*_ and wHC_*A*_) and global omnibus (i.e., MiHC) higher criticism tests (Fig. 2 (*n* = 50) and Additional file 4: Figure S3 (*n* = 100)); and (iii) the comparison for MiHC with the prior tests (i.e., Simes, HC, aMiAD, aMiSPU, and OMiRKAT) (Fig. 3 (*n* = 50) and Additional file 5: Figure S4 (*n* = 100)).

The individual (i.e., uHC_(*h*)_ and wHC_(*h*)_) tests are more powerful using a smaller *h* value for higher sparsity levels, while they are more powerful using a larger *h* value for lower sparsity levels (Fig. 1 and Additional file 3: Figure S2), which is explained by the modulation scheme (Eq. 8 and Eq. 9). The Simes test is powerful at high sparsity levels but rapidly loses power as the sparsity level decreases (Fig. 1 and Additional file 3: Figure S2). The Simes test is more powerful at high sparsity levels (i.e., ≤ 2% or ≤ 4%) even than the original high criticism test (i.e., uHC_(1)_), but it is less powerful at low sparsity levels (i.e., ≥ 4% or ≥ 6%) than any individual higher criticism tests (Fig. 1 and Additional file 3: Figure S2). uHC_*A*_ closely approaches the most powerful test among the individual unweighted tests (i.e., uHC_(*h*)_’s) and the Simes test (Fig. 1a, c and Additional file 3: Figure S2:A,C), while wHC_*A*_ closely approaches the most powerful test among the individual weighted tests (i.e., wHC_(*h*)_’s) and the Simes test (Fig. 1b, d and Additional file 3: Figure S2:B,D), which is explained by the adaptivity of the minimum *p* value statistic (Eq. 12 and Eq. 13). The unweighted tests (i.e., uHC_(*h*)_’s and uHC_*A*_) are more powerful than the weighted tests (i.e., wHC_(*h*)_’s and wHC_*A*_) when randomly selected OTUs are associated with the host phenotype (Fig. 1a > Fig. 1b, Additional file 3: Figure S2A > Additional file 3: Figure S2B, and Fig. 2a), while the weighted tests are more powerful than the unweighted tests when phylogenetically relevant OTUs are associated with the host phenotype (Fig. 1c < Fig. 1d, Additional file 3: Figure S2C < Additional file 3: Figure S2D, and Fig. 2b), which is explained by the weighting scheme for phylogenetic relevance (Eq. 7). In addition, the unweighted tests (i.e., uHC_(*h*)_’s and uHC_*A*_) are almost equally powerful when either randomly selected OTUs (Fig. 1a) or phylogenetically relevant OTUs (Fig. 1c) are associated with the host phenotype (Fig. 1a ≅ Fig. 1c). This is because the unweighted tests do not utilize any phylogenetic information; hence, they treat either randomly selected OTUs or phylogenetically relevant OTUs all equally as randomly selected OTUs.

To facilitate easier comparison, Fig. 2 and Additional file 4: Figure S3 report estimated powers only for the local omnibus (i.e., uHC_*A*_ and wHC_*A*_) and global omnibus (i.e., MiHC) higher criticism tests. Here again, uHC_*A*_ is more powerful than wHC_*A*_ when randomly selected OTUs are associated with the host phenotype (Fig. 2a and Additional file 4: Figure S3A), while wHC_*A*_ is more powerful than uHC_*A*_ when phylogenetically relevant OTUs are associated with the host phenotype (Fig. 2b and Additional file 4: Figure S3B), which is explained by the weighting scheme for phylogenetic relevance (Eq. 7). Importantly, we can observe that MiHC closely approaches the most powerful test between uHC_*A*_ and wHC_*A*_ (Fig. 2 and Additional file 4: Figure S3), which is explained by the adaptivity of the minimum *p* value statistic in the entirety (Eq. 11). This indicates that MiHC maintains a high power throughout different phylogenetic relevance and sparsity levels, while the individual or the local omnibus tests are limitedly powerful only for some specific phylogenetic relevance and sparsity levels (Figs. 1 and 2 and Additional files 3 and 4: Figure S2-S3). Thus, we suggest to use MiHC especially in respond to the unknown phylogenetic relevance and sparsity levels in practice.

Here, we also compare MiHC with the prior tests, Simes, HC, aMiAD, aMiSPU, and OMiRKAT. MiHC, Simes, and HC are powerful for high sparsity levels, while they lose power gradually for lower sparsity levels (Fig. 3 and Additional file 5: Figure S4). However, the power decay is slower for MiHC than Simes and HC (Fig. 3 and Additional file 5: Figure S4), which is explained by the modulation scheme (Eq. 8 and Eq. 9) and the adaptivity of the minimum *p* value statistic (Eq. 11). We can also observe that the power gap from MiHC to Simes or HC is larger when phylogenetically relevant OTUs are associated with the host phenotype (Fig. 3 and Additional file 5: Figure S4), which is explained by the weighting scheme for phylogenetic relevance (Eq. 7) and the adaptivity of the minimum *p* value statistic (Eq. 11). Therefore, MiHC better suits the microbiome association studies with multifarious phylogenetic relevance and sparsity levels. On the contrary, aMiAD, aMiSPU, and OMiRKAT are underpowered for high sparsity levels, yet they gain power gradually for lower sparsity levels (Fig. 3 and Additional file 5: Figure S4). This is because they amplify the overall group association by aggregating underlying microbial association signals in the sense of requiring as many association signals as possible. Especially, OMiRKAT is most powerful when phylogenetically relevant OTUs are associated with the host phenotype at low sparsity levels (i.e., ≥ 10%) (Fig. 3b), and we do not discourage the use of aMiAD, aMiSPU, and OMiRKAT for lower sparsity levels. MiHC is more powerful than aMiAD, aMiSPU, and OMiRKAT for many sparsity levels in our simulations (Fig. 3 and Additional file 5: Figure S4). We developed MiHC, from a different perspective, for the powerful discovery from high to low sparsity levels, which was especially challenging by the prior tests.

### Real data applications

#### The association between the respiratory tract microbiome and smoking status

Charlson et al. have collected swab samples from the upper respiratory tract to survey the effect of cigarette smoking on the respiratory tract microbiome [28]. The microbiome data for the OTU abundance table and phylogenetic tree are publicly available in the R package, *GUniFrac* [42], where the raw sequence data had been processed using the QIIME pipeline [2] by targeting the V1–2 region of the 16S ribosomal RNA (rRNA) gene (refer to [28] for more detailed sampling/data processing procedures) and the phylogenetic tree had been constructed by using FastTree [43, 44]. The microbiome data include 273 OTUs with mean relative abundance ≥ 10^−4^ for 60 samples (28 smokers and 32 non-smokers). Here, we test the association between respiratory tract microbial composition and smoking status while adjusting for gender and antibiotic use within the last 3 months.

**Fig. 4:**
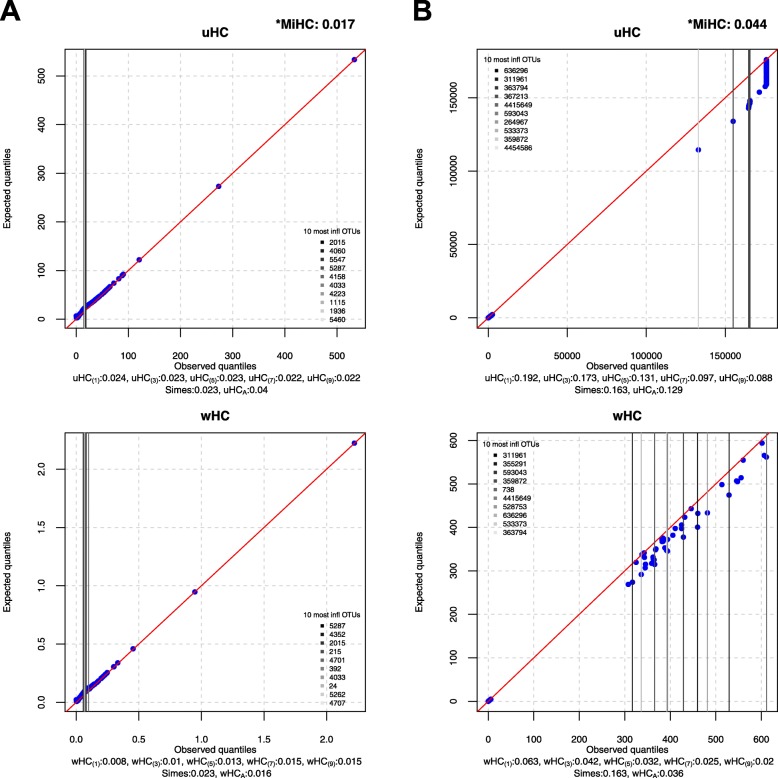
The Q-Q plots between the expected and observed quantiles for uHC and wHC, respectively. **a** The association between the respiratory tract microbiome and smoking status. **b** The association between the infant’s gut microbiome and delivery mode. Blue dots represent individual OTUs and a red diagonal line represents no influential points. Darker to lighter vertical lines represent more to less influential OTUs in rank order among the 10 most influential OTUs that correspond to the 10 largest deviations from the red diagonal line. The asterisk represents the *p* values for all the individual (i.e., uHC_(*h*)_’s and wHC_(*h*)_’s for *h* ∈ {1, 3, 5, 7, 9}) and omnibus (i.e., uHC_*A*_, wHC_*A*_, and MiHC) higher criticism tests and the Simes test

We found the significant association between respiratory tract microbial composition and smoking status throughout all the individual and omnibus higher criticism tests and the Simes test (Fig. 4a). We can also observe only a small difference between the unweighted (i.e., uHC_(*h*)_’s and uHC_*A*_) and weighted (i.e., wHC_(*h*)_’s and wHC_*A*_) tests (Fig. 4a), indicating that the OTUs associated with smoking status might have only mild phylogenetic relevance. We can also confirm it in visualization with similar graphical patterns between uHC and wHC (Fig. 4a). We also report the 10 most influential OTUs with respect to uHC and wHC, respectively (Fig. 4a). For the other competing methods, aMiAD and OMiRKAT find the significant association while aMiSPU does not (Table 2 A). MiHC finds the significant association (*p* value, 0.017) (Table 2 A and Fig. 4a).

**Table 2:** The *p* values for the individual (i.e., uHC_(*h*)_’s and wHC_(*h*)_’s for *h* ∈ {1, 3, 5, 7, 9}) and omnibus (i.e., uHC_*A*_, wHC_*A*_, and MiHC) higher criticism tests, the Simes test, and the other competing tests (i.e., aMiAD, aMiSPU, and OMiRKAT). (A) The association between respiratory tract microbiome and smoking status. (B) The association between the infant’s gut microbiome and delivery mode. (C) The association between the gut microbiome and type 1 diabetes status. (D) The association between the gut microbiome and human immunodeficiency virus status. * represents significant *p* values

Category		Method	A	B	C	D
Individual HC tests	Unweighted tests	uHC_(1)_	0.024*	0.192	0.025*	0.009*
		uHC_(3)_	0.023*	0.173	0.024*	0.008*
		uHC_(5)_	0.023*	0.131	0.024*	0.007*
		uHC_(7)_	0.022*	0.097	0.024*	0.007*
		uHC_(9)_	0.022*	0.088	0.024*	0.007*
	Weighted tests	wHC_(1)_	0.008*	0.063	0.026*	0.009*
		wHC_(3)_	0.010*	0.042*	0.024*	0.008*
		wHC_(5)_	0.013*	0.032*	0.023*	0.007*
		wHC_(7)_	0.015*	0.025*	0.023*	0.007*
		wHC_(9)_	0.015*	0.020*	0.024*	0.007*
Omnibus HC tests	Local omnibus tests	uHC_*A*_	0.040*	0.129	0.029*	0.013*
		wHC_*A*_	0.016*	0.036*	0.029*	0.012*
	Global omnibus tests	MiHC	0.017*	0.044*	0.030*	0.012*
Non-HC tests		Simes	0.023*	0.163	0.191	0.170
		aMiAD	0.035*	0.449	0.346	0.012*
		aMiSPU	0.063	0.170	0.017*	0.181
		OMiRKAT	0.005*	0.001*	0.016*	0.150

#### The association between the infant’s gut microbiome and delivery mode

Bokulich et al. have conducted a microbiome profiling study to survey the effect of the early life factors (e.g., delivery mode, infant nutrition, antibiotic use) on the infant’s gut microbiome [29]. As a demonstration, we test the association between the infant’s gut microbiome and delivery mode (i.e., vaginal or cesarean birth) while adjusting for gender and predominant diet (breastfeeding vs. formula). The microbiome data include 310 OTUs with mean relative abundance ≥ 10^−4^ for 32 infants (11 infants by cesarean delivery and 21 infants by vaginal delivery) [29, 37], where the raw sequence data had been processed using the QIIME pipeline [2] by targeting the V4 region of the 16S rRNA gene (refer to [29, 37] for more detailed sampling/data processing procedures) and the phylogenetic tree had been constructed by using FastTree [43, 44].

All the weighted tests (i.e., wHC_(*h*)_’s and wHC_*A*_) except for wHC_(1)_ found the significant association between the infant’s gut microbiome and delivery mode, while none of the unweighted tests (i.e., uHC_(*h*)_’s and uHC_*A*_) and the Simes test found it (Fig. 4b), indicating that the OTUs associated with delivery mode might have strong phylogenetic relevance. We can also confirm it in visualization with larger deviations between the expected and observed quantiles for wHC than uHC (Fig. 4b). We can also observe that the individual tests (i.e., uHC_(*h*)_ and wHC_(*h*)_) using a larger *h* value find smaller *p* values (Fig. 4b), indicating that many OTUs might be associated with delivery mode (i.e., low sparsity). We can also confirm it in visualization that many OTUs have some deviations between the expected and observed quantiles (Fig. 4b). We also report the 10 most influential OTUs with respect to uHC and wHC, respectively (Fig. 4b). For the other competing methods, OMiRKAT finds the significant association while aMiAD and aMiSPU do not (Table 2 B). MiHC finds the significant association (*p* value, 0.044) (Table 2 B and Fig. 4b).

#### The association between the gut microbiome and T1D status

Livanos et al. have conducted a microbiome profiling study to survey the roles of the gut microbiome on T1D onset through mouse experiments [30]. As a demonstration, we test the association between gut microbial composition and T1D status. For this, 19 mice were exposed to therapeutic-dose pulsed antibiotic (PAT) treatment at 6 weeks of age and then followed up for 30 weeks. The microbiome data include 120 OTUs with mean relative abundance ≥ 10^−4^ for the 19 mice at 30 weeks of the follow-up (9 T1D-free mice and 10 T1D-onset mice), where the raw sequence data had been processed using the QIIME pipeline [2] by targeting the V4 region of the 16S rRNA gene (refer to [30] for more detailed sampling/data processing procedures) and the phylogenetic tree had been constructed by using FastTree [43, 44].

**Fig. 5:**
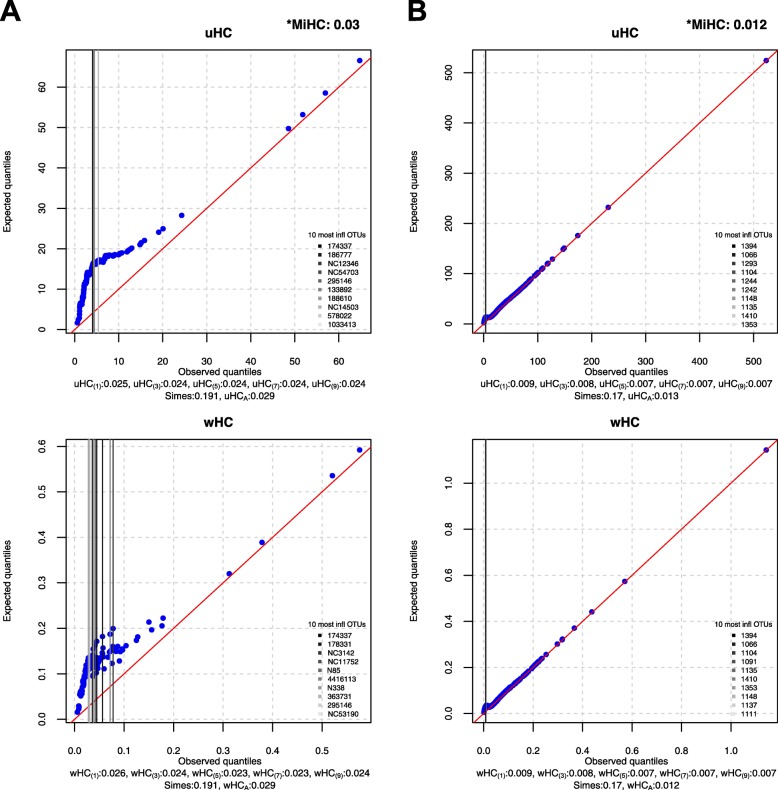
The Q-Q plots between the expected and observed quantiles for uHC and wHC, respectively. **a** The association between the gut microbiome and type 1 diabetes status. **b** The association between the gut microbiome and human immunodeficiency virus status. Blue dots represent individual OTUs and a red diagonal line represents no influential points. Darker to lighter vertical lines represent more to less influential OTUs in rank order among the 10 most influential OTUs that correspond to the 10 largest deviations from the red diagonal line. The asterisk represents the *p* values for all the individual (i.e., uHC_(*h*)_’s and wHC_(*h*)_’s for *h* ∈ {1, 3, 5, 7, 9}) and omnibus (i.e., uHC_*A*_, wHC_*A*_, and MiHC) higher criticism tests and the Simes test

We found the significant association between gut microbial composition and T1D status throughout all the individual and omnibus higher criticism tests but not through the Simes test (Fig. 5a). We can also observe only a small difference between the unweighted (i.e., uHC_(*h*)_’s and uHC_*A*_) and weighted (i.e., wHC_(*h*)_’s and wHC_*A*_) tests (Fig. 5a). This might indicate that the OTUs associated with T1D status have only mild phylogenetic relevance. We also report the 10 most influential OTUs with respect to uHC and wHC, respectively (Fig. 5a). For the other competing methods, aMiSPU and OMiRKAT find the significant association while aMiAD does not (Table 2 C). MiHC finds the significant association (*p* value, 0.03) (Table 2 C and Fig. 5a).

#### The association between the gut microbiome and HIV status

Pinto-Cardoso et al. have conducted a microbiome profiling study to survey the effect of antiretroviral therapy (ART) on the gut microbiome of HIV-positive individuals [31]. As a demonstration, we test the association between gut microbial composition and HIV status while adjusting for age. For this, 33 HIV-infected individuals on ART and 10 HIV-uninfected individuals from Mexico were included in the analysis [31]. The microbiome data include 422 OTUs with mean relative abundance ≥ 10^−4^ for the 44 individuals, where the raw sequence data had been processed using the Resphera Insight [45] by targeting the V3 and V4 regions of the 16S rRNA gene (refer to [31, 46] for more detailed sampling/data processing procedures) and the phylogenetic tree had been constructed by using PyNAST [47].

We found the significant association between gut microbial composition and HIV status throughout all the individual and omnibus higher criticism tests, but not through the Simes test (Fig. 5b). We can also observe only a small difference between the unweighted (i.e., uHC_(*h*)_’s and uHC_*A*_) and weighted (i.e., wHC_(*h*)_’s and wHC_*A*_) tests (Fig. 5b), indicating that the OTUs associated with T1D status might have only mild phylogenetic relevance. We can also confirm it in visualization with similar graphical patterns between uHC and wHC (Fig. 5b). We also report the 10 most influential OTUs with respect to uHC and wHC, respectively (Fig. 5b). For the other competing methods, aMiAD finds the significant association while aMiSPU and OMiRKAT do not (Table 2 D). MiHC finds the significant association (*p* value, 0.012) (Table 2 D and Fig. 5b).

## Discussion and conclusions

In this paper, we introduced a data-driven omnibus test, MiHC, to evaluate the association between microbial group (e.g., community or clade) composition and a host phenotype of interest. Our simulations demonstrated that MiHC robustly maintains a high power for both random and phylogenetic association patterns at different high sparsity levels with correct type I error controls. We also applied MiHC to four different real microbiome datasets and observed that MiHC finds stably low *p* values while the individual (i.e., uHC_(*h*)_’s and wHC_(*h*)_’s) higher criticism tests and the Simes test find differing *p* values depending on the underlying phylogenetic relevance and sparsity levels. Thus, MiHC is a useful analytic tool in practice because of the unknown phylogenetic relevance and sparsity levels.

We considered the optimal number of clusters which maximizes the average silhouette width searching up to 30 clusters and the candidate set of Г = {1, 3, 5, 7, 9}, instead of the union set of Г = {1, 2,…, *m*-1, *m*}, for the individual (i.e., uHC_(*h*)_’s and wHC_(*h*)_’s) higher criticism tests to avoid the exhaustive search and huge computation. However, any other upper limit to fine the optimal number of clusters and any other smaller or larger candidate set can alternatively be considered by the researcher’s choice through the options in our software package. For example, you may believe that the candidate set of Г = {1, 3, 5, 7, 9} is too much tailored to high sparsity levels; hence, you can include larger values in the candidate set for lower sparsity levels. Moreover, a number of microbiome data normalization procedures have been proposed [48], but there is no consensus on which procedure is the best and such debate is beyond the scope of this paper. We did not survey any further normalization procedure except for using relative abundances (i.e., proportions), instead of absolute abundances (i.e., read counts), to control differing total read counts per sample. However, MiHC is compatible with any other normalization procedure (e.g., centered log-ratio transformation [49]), which can be considered by the researcher’s choice. We set up all the implementation procedures described in this paper as a default in our software package, yet we do not strictly force to use it. Instead, we give researches some user options in our software package to make the best use of it.

We developed MiHC based on the generalized linear models to handle exponential family responses with the linear predictor [32]. However, its application can be much broader, and, for example, the potential extensions to survival [38, 50], longitudinal [39, 51], or mediation [52] analysis need to be further studied.

## Supplementary information

**Additional file 1: Table S1.** The summary of the notations.

**Additional file 2: Figure S1.** The histograms of the relative abundances (%). A. The real respiratory-track microbiome data. B. The simulated data based on the Dirichlet-multinomial model (*n*=50). C. The simulated data based on the Dirichlet-multinomial model (*n*=100).

**Additional file 3: Figure S2.** Power estimates for the individual (i.e., uHC_(*h*)_’s and wHC_(*h*)_’s for *h* ∈ {1, 3, 5, 7, 9}) and local omnibus (uHC_*A*_ and wHC_*A*_) higher criticism tests (*n*=100) (Unit: %). A. uHC_(*h*)_’s and uHC_*A*_ for the randomly selected OTUs (i.e., Λ = {2%, 4%, 6%, 8%, 10% or 12% random OTUs}). B. wHC_(*h*)_’s and wHC_*A*_ for the phylogenetically relevant OTUs (i.e., Λ = {2%, 4%, 6%, 8%, 10% or 12% phylogenetically close OTUs}). C. uHC_(*h*)_’s and uHC_*A*_ for the randomly selected OTUs (i.e., Λ = {2%, 4%, 6%, 8%, 10% or 12% random OTUs}). D. wHC_(*h*)_’s and wHC_*A*_ for the phylogenetically relevant OTUs (i.e., Λ = {2%, 4%, 6%, 8%, 10% or 12% phylogenetically close OTUs}).

**Additional file 4: Figure S3.** Power estimates for the omnibus (uHC_*A*_, wHC_*A*_ and MiHC) higher criticism tests (*n*=100) (Unit: %). A. For the randomly selected OTUs (i.e., Λ = {2%, 4%, 6%, 8%, 10% or 12% random OTUs}. B. For the phylogenetically relevant OTUs (i.e., Λ = {2%, 4%, 6%, 8%, 10% or 12% phylogenetically close OTUs}).

**Additional file 5: Figure S4.** Power estimates for MiHC compared with the prior tests, HC, aMiAD, aMiSPU and OMiRKAT (*n*=100) (Unit: %). A. For the randomly selected OTUs (i.e., Λ = {2%, 4%, 6%, 8%, 10% or 12% random OTUs}. B. For the phylogenetically relevant OTUs (i.e., Λ = {2%, 4%, 6%, 8%, 10% or 12% phylogenetically close OTUs}).

## Data Availability

We used four public microbiome datasets for (1) the association between respiratory tract microbiome and smoking status (available in the R package, *GUniFrac*, with three data objects, throat.otu.table, throat.tree, and throat.meta, https://cran.r-project.org/web/packages/GUniFrac/index.html), (2) the association between the infant’s gut microbiome and delivery mode (available in the QIITA repository under accession code 10249, https://qiita.ucsd.edu), (3) the association between the gut microbiome and T1D status (available in the European Bioinformatics Institute (EBI) database under accession code ERP016357, https://www.ebi.ac.uk and in the Qiita database under accession code: 10508, https://qiita.ucsd.edu), and (4) The association between the gut microbiome and HIV status (available in the European Bioinformatics Institute (EBI) database under accession code PRJNA344791, https://www.ebi.ac.uk). Our propose method can be implemented using the R package, MiHC, which is freely available at https://github.com/hk1785/MiHC. The detailed manual on the inputs, outputs, arguments, and options can also be found in the software webpage.

## Abbreviations

aMiAD: Adaptive Microbiome *α*-diversity-based association test
aMiSPU: Adaptive microbiome sum of powered score tests
ART: Antiretroviral therapy
HC: Higher criticism
HIV: Human immunodeficiency virus
MiHC: Microbiome higher criticism analysis
MiRKAT: Microbiome regression-based kernel association test
MiSPU: Microbiome sum of powered score tests
OMiRKAT: Optimal microbiome regression-based kernel association test
OTU: Operational taxonomic unit
RNA: Ribosomal RNA
T1D: Type I diabetes
uHC: Unweighted higher criticism
wHC: Weighted higher criticism
