# Supplementary material for: A powerful microbial group association test based on the higher criticism analysis for sparse microbial association signals
Source: Microbiome. 2020 May 11;8:63. doi: 10.1186/s40168-020-00834-9 (PMC7216722; doi:10.1186/s40168-020-00834-9)
Supplement: Supplementary file 1 — Additional file 1: Table S1. The summary of the notations. [file 40168_2020_834_MOESM1_ESM.pdf]

Table S1. The summary of the notations.

Notation	Description
n	The number of samples
m	The number of OTUs in a microbial group of interest (e.g., community or clade)
l	The number of covariates (e.g., age, gender)
i	The subscript for a sample, where $i = 1, \ldots, n$
j	The subscript for an OTU, where $j = 1, \ldots, m$
k	The subscript for a covariate, where $k = 1, \ldots, l$
y_i	An element of the host phenotype of the i-th sample
x_{ik}	An element of the covariate of the i-th sample and the k-th covariate
o_{ij}	An element of the OTU in relative abundance of the i-th sample and the j-th OTU
$g(\cdot)$	A canonical link function of the GLM (Eq. 1)
x_i	An $(l+1) \times 1$ vector of the covariates; $x_i = (1, x_{i1}, \ldots, x_{il})^T$
o_i	An $m \times 1$ vector of the OTUs in relative abundance; $o_i = (o_{i1}, \ldots, o_{im})^T$
μ_i	The expected value of y_i's given x_i and o_i of the GLM (Eq. 1); $\mu_i = E(y_i \| x_i, o_i)$
α_k	An element of the regression coefficient for the k-th covariate of the GLM (Eq. 1)
β_j	An element of the regression coefficient for the j-th OTU of the GLM (Eq. 1)
α	An $(l+1) \times 1$ vector of the regression coefficients for the covariates of the GLM (Eq. 1); $\alpha = (\alpha_0, \ldots, \alpha_l)^T$
β	An $m \times 1$ vector of the regression coefficients for the OTUs of the GLM (Eq. 1); $\beta = (\beta_1, \ldots, \beta_m)^T$
θ_i, φ	The natural and dispersion parameters of the GLM (Eq. 1-2)
$a(\cdot)$, $b(\cdot)$, $c(\cdot)$	The known functions of the GLM (Eq. 1-2)
o_j	An $n \times 1$ vector of the OTUs in relative abundance; $o_j = (o_{1j}, \ldots, o_{nj})^T$
y	An $n \times 1$ vector of the host phenotypes; $y = (y_1, \ldots, y_n)^T$
$\hat{\mu}_0$	An $n \times 1$ vector of the expected values of y_i's under the null model of $g(\mu_i) = x_i^T\alpha$; $\hat{\mu}_0 = (\hat{\mu}_{1,0}, \ldots, \hat{\mu}_{n,0})^T = (g^{-1}(X_1^T\hat{\alpha}_0), \ldots, g^{-1}(X_n^T\hat{\alpha}_0))^T = (b'(\hat{\theta}_{1,0}), \ldots, b'(\hat{\theta}_{n,0}))^T$
W	An $n \times n$ diagonal matrix of the marginal variances of y_i's under the null model of $g(\mu_i) = x_i^T\alpha$; $W = diag(a_1(\hat{\varphi}_0)b''(\hat{\theta}_{1,0}), \ldots, a_n(\hat{\varphi}_0)b''(\hat{\theta}_{n,0}))$
X	An $n \times (l+1)$ matrix of the covariates; $X = (x_1, \ldots, x_n)^T$
P	An $n \times n$ matrix; $P = W - WX(X^TWX)^{-1}X^TW$
Z_j	The marginal standardized score statistic for the j-th OTU (Eq. 4)
p_j	The p-value for the j-th OTU
r_j	The rank of p_j in the ascending order of p_j's for $j = 1, \ldots, m$
$D_{j,j'}$	The cophenetic distance between j-th and j'-th OTUs
C	The optimal number of clusters which maximized the average silhouette width
$\zeta(j)$	The cluster anchored at the j-th OTU among the C clusters
w_j	The weight for the j-th OTU (Eq. 7)
$\frac{r_{j'}/m - p_{j'}}{\sqrt{p_{j'}(1-p_{j'})/m}}$	The j'-th order statistics of $\frac{r_j/m - p_j}{\sqrt{p_j(1-p_j)/m}}$'s in the descending order for $j = 1, \ldots, m$
$\frac{w_{j'}(r_{j'}/m - p_{j'})}{\sqrt{p_{j'}(1-p_{j'})/m}}$	The j'-th order statistics of $\frac{w_j(r_j/m - p_j)}{\sqrt{p_j(1-p_j)/m}}$'s in the descending order for $j = 1, \ldots, m$
h	A pre-specified number; $h \in \{1, 2, \ldots, m\text{-}1, m\}$
$\text{uHC}_{(h)}$, $P_{uHC(h)}$	The test statistic and p-value of the unweighted higher criticism test for a given h value (Eq. 8)
$\text{wHC}_{(h)}$, $P_{wHC(h)}$	The test statistic and p-value of the weighted higher criticism test for a given h value (Eq. 9)
T_{Simes}, P_{Simes}	The test statistic and p-value of the Simes test (Eq. 10)
T_{MiHC}, P_{MiHC}	The test statistic and p-value of the global omnibus test MiHC (Eq. 11)
T_{uHC_A}, P_{uHC_A}	The test statistic and p-value of the local omnibus test uHC_A (Eq. 12)
T_{wHC_A}, P_{wHC_A}	The test statistic and p-value of the local omnibus test wHC_A (Eq. 13)
