# Supplementary figures and images for: A powerful microbial group association test based on the higher criticism analysis for sparse microbial association signals
Source: Microbiome. 2020 May 11;8:63. doi: 10.1186/s40168-020-00834-9 (PMC7216722; doi:10.1186/s40168-020-00834-9)

**A**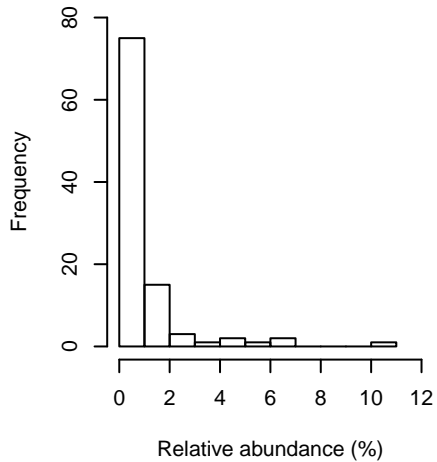**B**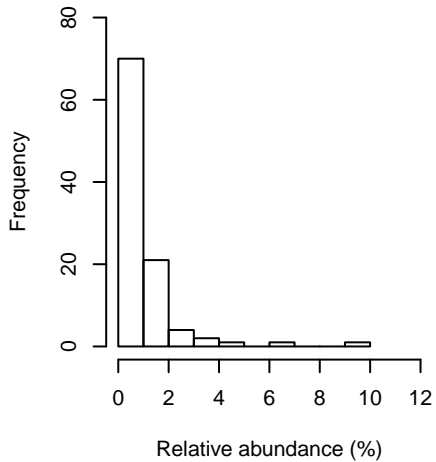**C**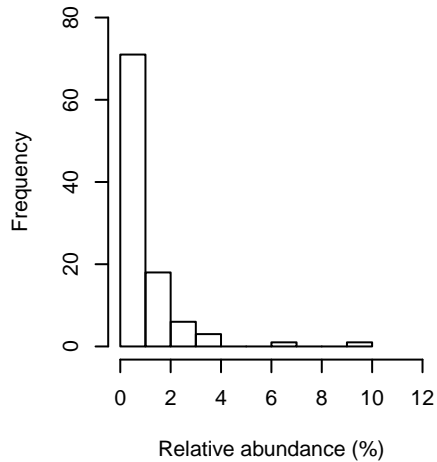

Supplement: Supplementary file 2 — Additional file 2: Figure S1. The histograms of the relative abundances (%). A. The real respiratory-track microbiome data. B. The simulated data based on the Dirichlet-multinomial model (n=50). C. The simulated data based on the Dirichlet-multinomial model (n=100). [file 40168_2020_834_MOESM2_ESM.pdf]

**A**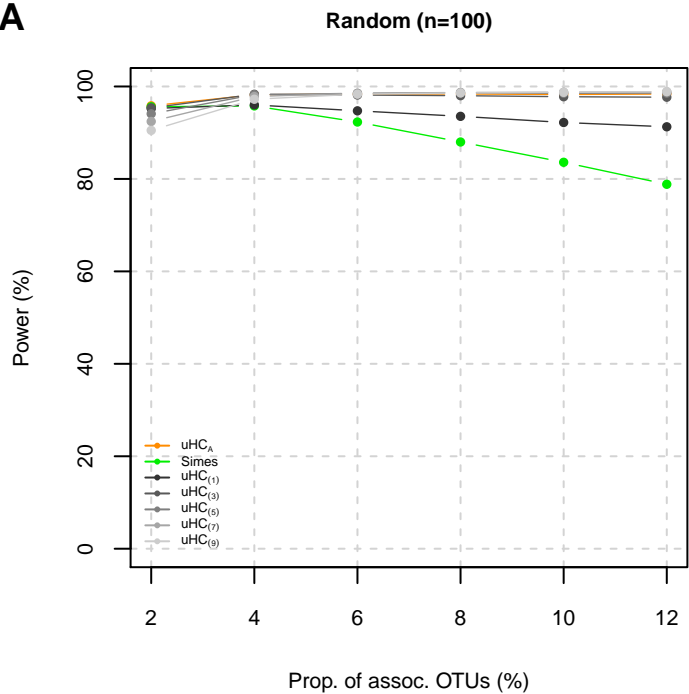**B**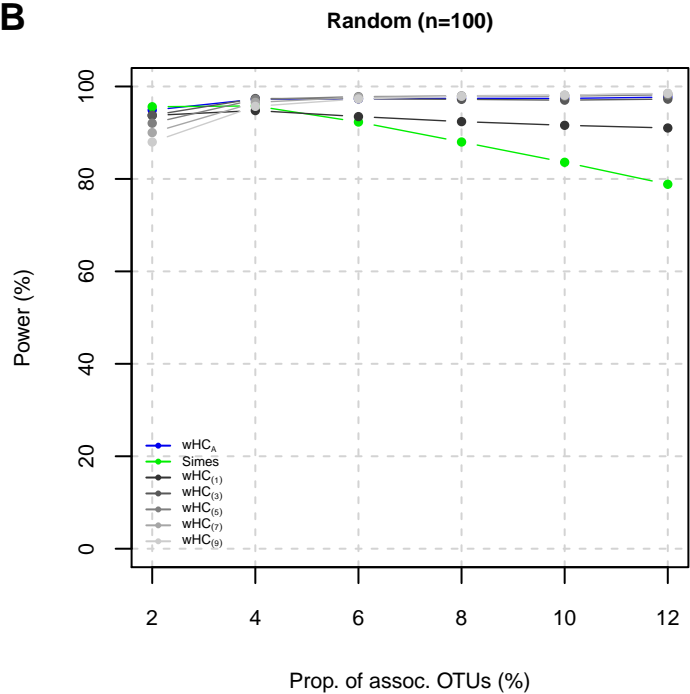**C**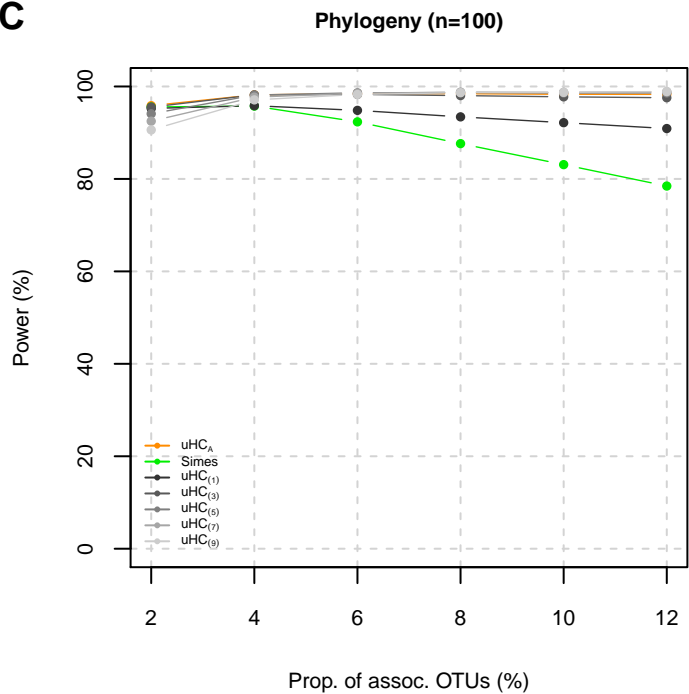**D**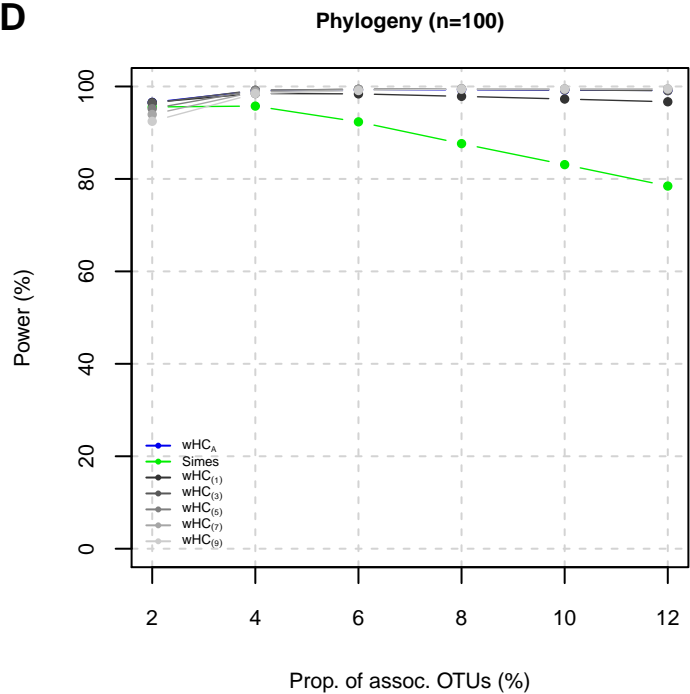

Supplement: Supplementary file 3 — Additional file 3: Figure S2. Power estimates for the individual (i.e., uHC(h)’s and wHC(h)’s for h ∈ {1, 3, 5, 7, 9}) and local omnibus (uHCA and wHCA) higher criticism tests (n=100) (Unit: %). A. uHC(h)’s and uHCA for the randomly selected OTUs (i.e., Λ = {2%, 4%, 6%, 8%, 10% or 12% random OTUs}). B. wHC(h)’s and wHCA for the phylogenetically relevant OTUs (i.e., Λ = {2%, 4%, 6%, 8%, 10% or 12% phylogenetically close OTUs}). C. uHC(h)’s and uHCA for the randomly selected OTUs (i.e., Λ = {2%, 4%, 6%, 8%, 10% or 12% random OTUs}). D. wHC(h)’s and wHCA for the phylogenetically relevant OTUs (i.e., Λ = {2%, 4%, 6%, 8%, 10% or 12% phylogenetically close OTUs}). [file 40168_2020_834_MOESM3_ESM.pdf]

**A****Random (n=100)**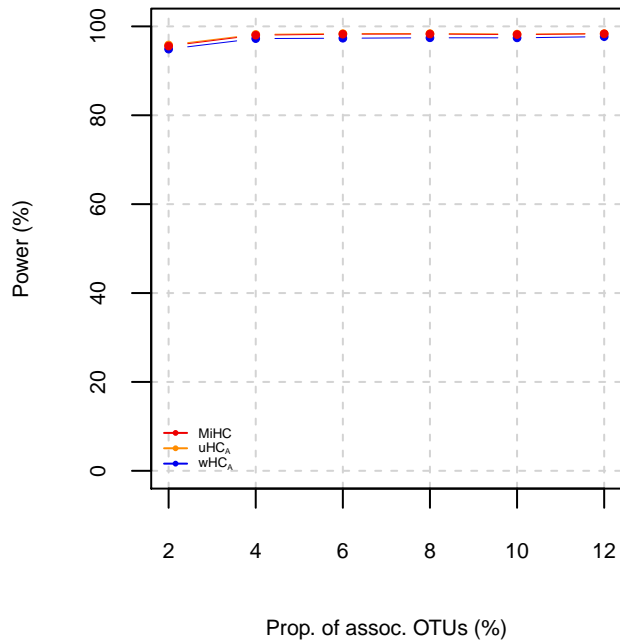**B****Phylogeny (n=100)**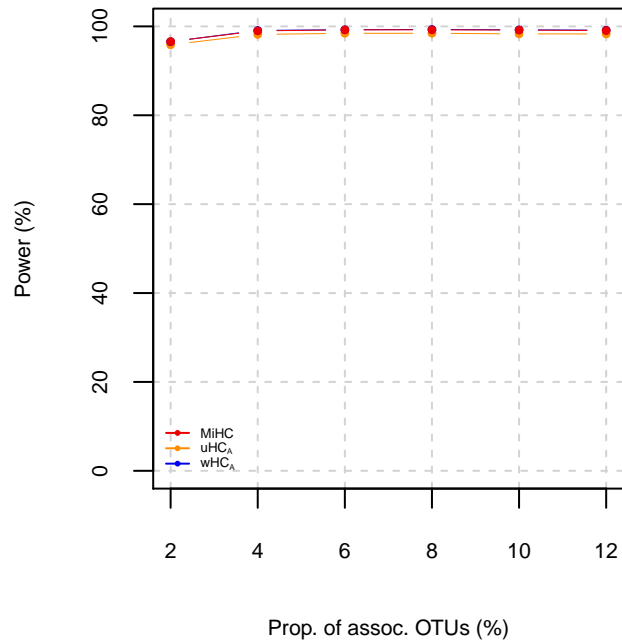

Supplement: Supplementary file 4 — Additional file 4: Figure S3. Power estimates for the omnibus (uHCA, wHCA and MiHC) higher criticism tests (n=100) (Unit: %). A. For the randomly selected OTUs (i.e., Λ = {2%, 4%, 6%, 8%, 10% or 12% random OTUs}. B. For the phylogenetically relevant OTUs (i.e., Λ = {2%, 4%, 6%, 8%, 10% or 12% phylogenetically close OTUs}). [file 40168_2020_834_MOESM4_ESM.pdf]

**A****Random (n=100)**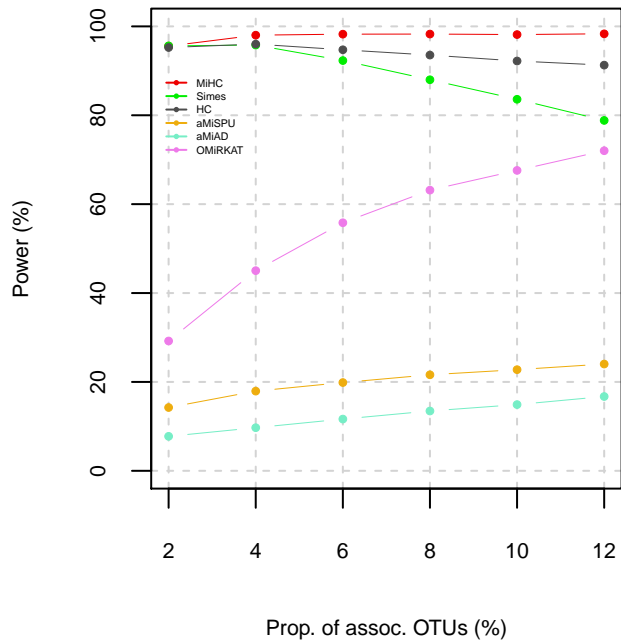**B****Phylogeny (n=100)**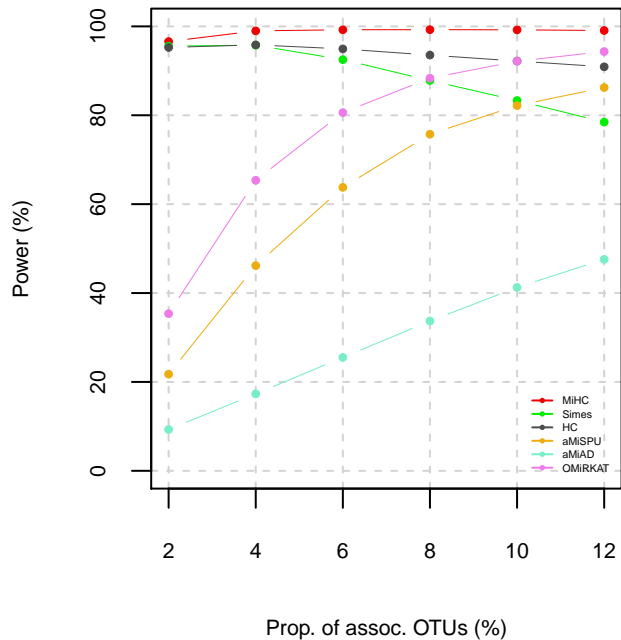

Supplement: Supplementary file 5 — Additional file 5: Figure S4. Power estimates for MiHC compared with the prior tests, HC, aMiAD, aMiSPU and OMiRKAT (n=100) (Unit: %). A. For the randomly selected OTUs (i.e., Λ = {2%, 4%, 6%, 8%, 10% or 12% random OTUs}. B. For the phylogenetically relevant OTUs (i.e., Λ = {2%, 4%, 6%, 8%, 10% or 12% phylogenetically close OTUs}). [file 40168_2020_834_MOESM5_ESM.pdf]
